# Contrast-Enhanced Ultrasound Imaging of Uterine Disorders: A Systematic Review

**DOI:** 10.1177/01617346211017462

**Published:** 2021-05-26

**Authors:** Barbara Stoelinga, Lynda Juffermans, Anniek Dooper, Marleen de Lange, Wouter Hehenkamp, Thierry Van den Bosch, Judith Huirne

**Affiliations:** 1 Amsterdam UMC Locatie VUmc, Amsterdam, The Netherlands; 2 Amsterdam UMC Locatie Meibergdreef, Amsterdam, The Netherlands; 3 Katholieke Universiteit Leuven UZ Leuven, Leuven, Belgium; 4 Amsterdam UMC Locatie De Boelelaan, Amsterdam, The Netherlands

**Keywords:** contrast-enhanced ultrasound, imaging, microvasculature, uterine disorders, systematic review

## Abstract

Uterine disorders are often presented with overlapping symptoms. The microvasculature holds specific information important for diagnosing uterine disorders. Conventional sonography is an established diagnostic technique in gynecology, but is limited by its inability to image the microvasculature. Contrast-enhanced ultrasound (CEUS), is capable of imaging the microvasculature by means of intravascular contrast agents; that is, gas-filled microbubbles. We provide a literature overview on the use of CEUS in diagnosing myometrial and endometrial disorders, that is, fibroids, adenomyosis, leiomyosarcomas and endometrial carcinomas, as well as for monitoring and enhancing the effectiveness of minimally invasive therapies. A systematic literature search with quality assessment was performed until December 2020. In total 34 studies were included, published between 2007 and 2020.The results entail a description of contrast-enhancement patterns obtained from healthy tissue and from malignant and benign tissue; providing a first base for potential diagnostic differentiation in gynecology. In addition it is also possible to determine the degree of myometrial invasion in case of endometrial carcinoma using CEUS. The effectiveness of minimally invasive therapies for uterine disorders can safely and accurately be assessed with CEUS. In conclusion, the abovementioned applications of CEUS are promising and it is worth further exploring its full potential for gynecology by designing innovative and methodologically high-quality clinical studies.

## Introduction

Uterine disorders are often presented with overlapping symptoms. Some disorders, such as fibroids, are usually correctly identified by conventional grayscale or Doppler imaging. Other disorders such as diffuse adenomyosis and malignant leiomyosarcomas are often difficult to distinguish from common fibroids. The microvascular architecture and blood flow patterns of uterine disorders can provide detailed information important for accurate differentiation. However, detection of vessels smaller than 0.1 mm in diameter and full quantification of vascular flow remain beyond the reach of conventional grayscale and Doppler imaging techniques.^1^

**Figure 1.**
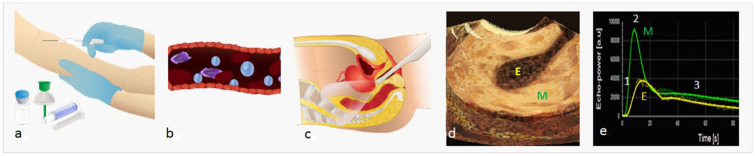
Schematic representation of performing contrast-enhanced ultrasonography (CEUS) in gynecological setting. *Step 1*: intravenous injection of, for example, SonoVue ultrasound contrast agent. Injection is regulary done via a catheter and 5 mL of saline is flushed after the diluted sonovue is injected (a). Ultrasound contrast agents (microbubbles) remain intravascular (b). *Step 2*: transvaginal (or abdominal) ultrasound scan of the uterus (c). Providing a contrast-enhanced image of the uterus (d), showing hyper-enhancement of the myometrium (M) compared with the endometrium (E). *Step 3*: the CEUS image can be fully quantified, providing a time-intensity curve (e) from which parameters such as wash-in rate (1), peak intensity (2), and wash-out rate (3) can be obtained.

Contrast-enhanced ultrasound (CEUS), not to be confused with saline- or gel-infused sonohysterography, is an imaging technique capable of visualizing both macro- and microvasculature by means of intravenously injected gas-filled microbubbles.^2^ A schematic overview for performing CEUS in gynecological setting is depicted in Figure 1. Microbubbles oscillate in the ultrasound beam, thereby reflecting a unique, non-linear echo that stands in contrast to the linear echo produced by the surrounding tissue. This non-linear echo, containing harmonics of the transmitted frequency, can be converted into a contrast-enhanced image that displays tissue vascularization, as microbubbles remain intravascular.^3^

Another important advantage of CEUS is the possibility of full quantification of the signal. Quantification of the contrast-enhanced signal is mostly established by analyzing the tissue-specific parameters, such as wash-in phase, peak intensity and wash-out phase, which are converted into a time-intensity curve (Figure 1(e)). The wash-in phase starts from the first arrival of contrast, usually 20 to 30 seconds after intravenous injection, during this period the level of intensity increases progressively until it reaches a plateau (peak intensity). This is followed by the wash-out phase during which the signal disappears or falls into noise level. Research often focusses on finding one parameter or a combination of parameters that is specific for discrimination for example malignant from benign tissue.

In general, the microvasculature of benign tumor tissue is different from malignant tumor tissue. Malignant lesions show enhanced tumor-induced angiogenesis, that is, sprouting of new blood vessels enabling tumor growth. These new tumor vessels are generally disorganized and leaky, often with incomplete vessel wall musculature and larger diameter resulting in low resistance to flow.^4,5^ Imaging the microvasculature of uterine abnormalities with CEUS may allow for differentiation between benign and malignant uterine disorders.

Besides using CEUS for diagnosing uterine disorders, it can be applied to monitor efficacy of minimally invasive treatments, such as ablation (high-intensity focused ultrasound (HIFU) or microwave ablation) or vascular occlusion (uterine artery embolization). The success rate of minimally invasive treatments, such as ablation or vascular occlusion, relies on the reduction of blood flow. The degree of success can be monitored by the degree of shrinkage or improvement of symptoms. However, there is a delay between treatment and observing shrinkage and/or reduction of symptoms. CEUS could be of prognostic value here, as there is a strong correlation between the degree of vascularity of a fibroid and the success rate of vascular occlusion.^6^ For ablation therapy the opposite applies, higher vascularity predicts poor ablation efficacy.^7^

CEUS is already an established clinical technique for assessing liver lesions,^3,8^ renal carcinoma,^9,10^ and in cardiac imaging,^11^ but its value is still to be demonstrated in assessing gynecological disorders. The high potential of CEUS in gynecology was mentioned already in 1997 by Abramowicz.^12^. In 2005 he wrote “Ultrasonographic contrast media: has the time come in obstetrics and gynecology.”^13^ Another decade later Pop et al.^14^ posed the application of CEUS for diagnosing endometrial pathologies, but concluded that more prospective studies are needed to reach an established role for CEUS in gynecology. The use of CEUS in gynecology was added to the latest update (2018) of the European Federation of Societies for Ultrasound in Medicine and Biology (EFSUMB) guidelines for CEUS in non-hepatic applications,^15^ illustrating the novelty of CEUS in this field.

This systematic review provides a literature overview on the potential use of CEUS in diagnosing myometrial and endometrial disorders, differentiating benign and malignant lesions, as well as for monitoring and enhancing effectiveness of minimally invasive therapies. A description of contrast-enhancement characteristics of different types of tissue is reported, dependent on the description provided in the included literature.

## Materials and Methods

This systematic review was conducted in accordance with PRISMA (Preferred Reporting Items for Systematic Review and Meta-Analyses) guidelines. In December 2020 one author (BS) performed the search with assistance of a medical science librarian of the Amsterdam UMC in PubMed, Embase, and Cochrane computerized bibliographic databases for eligible studies on patients with myometrial and endometrial disorders (fibroids, adenomyosis, uterine polyps, endometrial cancer, and leiomyosarcoma) who underwent a CEUS for diagnostic purpose or for monitoring minimal invasive therapy. The details of the search strategy are presented in Supplemental Appendix A. Cohort studies, case-control studies, and systematic reviews, published as full paper in English peer-reviewed journals were eligible for inclusion. Studies with less than 10 patients, letters and conference abstracts were excluded, as well as pre-clinical studies about CEUS in animals or laboratory models. Studies reporting on CEUS in diagnosing uterine pathology had to use a reference test. Studies reporting on monitoring effectiveness of minimally invasive therapies were also included when using MRI or conventional US besides CEUS, but without a reference test such as histology. The primary outcome was CEUS characteristics, including enhancement pattern, blood supply, and quantification of perfusion parameters.

After removal of duplicates, two authors (BS and AD) independently screened titles and abstracts to select articles meeting the eligibility criteria. Full texts of the remaining papers were assessed, discrepancies were resolved by discussion. The following data was extracted by BS and AD independently: study design, number of patients, study period, target condition, reference test and outcome measurements.

The risk of bias and methodological quality of the selected studies was assessed (BS and AD) using the quality assessment of diagnostic accuracy studies (QUADAS)-2 tool.^16^ Disagreement was resolved by discussion with a third researcher (JH). The QUADAS tool consists of four key domains: *patient selection*: describing how the patient population was selected; *index test*: describing how the test was conducted; *reference standard*: describing how the reference standard was conducted and *flow/timing*: describing the flow of patient inclusion and exclusion and the interval between the index test and reference standard. The four domains are assessed for risk of bias and rated as a low, high or unclear risk. The first three domains are also assessed for applicability concerns (does the study match the review question?).

When available, data on diagnostic accuracy, sensitivity, specificity, negative predictive value (NPV), positive predictive value (PPV), interclass correlation coefficient (ICC) was extracted or re-calculated by the authors of this review. Although the EFSUMB provided guidelines on how to describe enhancement characteristics in a consistent manner,^15^ this is not consistently applied by the cited manuscripts. Enhancement characteristics and quantitative parameters are therefore reported in this review as described in the cited article.

## Results

### Literature Identification

**Figure 2.**
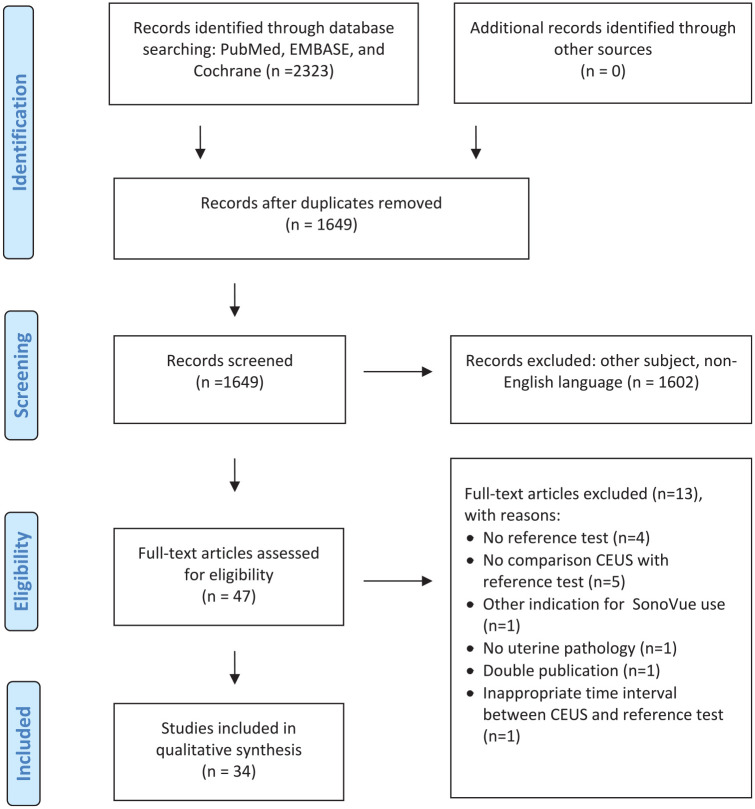
Flow chart of literature search on articles reporting on CEUS for diagnosis of uterine disorder, monitoring, and/or enhancing of minimally invasive therapy.

Searching PubMed, EMBASE, and Cochrane yielded 2323 records (Figure 2). After removing duplicates, title and abstract of 1649 records were screened, resulting in 47 articles. After full-text assessment another 13 articles were excluded. Finally, a total of 34 articles from four countries were included (26 from China). All studies were published between 2007 and 2021. Methodological quality assessment showed a limited number of methodological high-quality studies (Table 1), indicating that results and conclusions should be interpreted with care, keeping bias in mind.

**Table 1.** Studies included in review according to the Quality Assessment of Diagnostic Accuracy Studies-2 (QUADAS-2).

	Risk of BIAS				Applicability concerns		
	Patient selection	Index test	Reference standard	Flow and timing	Patient selection	Index test	Reference standard
Diagnosis by CEUS							
Liu et al.^17^	**H**	**L**	**L**	**H**	**L**	**L**	**L**
Zhang et al.^5^	**H**	**L**	**H**	**H**	**L**	**L**	**L**
Song et al.^18^	**H**	**H**	**L**	**H**	**L**	**L**	**L**
Lieng et al.^19^	**L**	**L**	**H**	**H**	**L**	**L**	**L**
Liu et al.^20^	**H**	**L**	**L**	**L**	**L**	**L**	**L**
Zhou et al.^21^	**H**	**L**	**L**	**L**	**L**	**L**	**L**
Liu et al.^22^	**L**	**L**	**L**	**L**	**L**	**L**	**L**
Su et al.^23^	**H**	**L**	**L**	**L**	**L**	**L**	**L**
Zhang et al.^24^	**H**	**L**	**L**	**L**	**L**	**L**	**L**
Green and Epstein^25^	**H**	**L**	**L**	**L**	**L**	**L**	**L**
Li et al.^26^	**H**	**L**	**H**	**H**	**L**	**L**	**L**
CEUS monitoring effectiveness of therapies							
Dorenberg et al.^27^	**L**	**L**	**H**	**L**	**L**	**L**	**L**
Zhou et al.^28^	**H**	**H**	**H**	**L**	**L**	**L**	**L**
Sconfienza et al.^29^	**H**	**H**	**L**	**H**	**L**	**L**	**L**
Wang et al.^30^	**L**	**H**	NA	**L**	**L**	**L**	NA
Wang et al.^31^	**H**	**L**	NA	**H**	**L**	**L**	NA
Wang et al.^32^	**H**	**H**	NA	**H**	**L**	**L**	NA
Lei et al.^33^	**H**	**L**	**H**	**L**	**L**	**L**	**L**
Henri et al.^34^	**L**	**L**	**H**	**L**	**L**	**L**	**L**
Wang et al.^35^	**L**	**H**	**H**	**L**	**L**	**L**	**L**
Xia et al.^36^	**L**	**L**	NA	**L**	**L**	**L**	NA
Yu et al.^37^	**L**	**L**	**L**	**H**	**L**	**L**	**L**
Zhang et al.^38^	**L**	**L**	**L**	**H**	**L**	**L**	**L**
Wang et al.^39^	**H**	**L**	**L**	**L**	**L**	**L**	**L**
Xu et al.^40^	**H**	**L**	**L**	**L**	**L**	**L**	**L**
CEUS-Enhancing effectiveness HIFU							
Peng et al.^41^	**H**	**H**	**L**	**H**	**L**	**L**	**L**
Dorenberg et al.^42^	**H**	**H**	**H**	**L**	**L**	**L**	**L**
Cheng et al.^43^	**L**	**L**	**L**	**L**	**L**	**L**	**L**
Isern et al.^44^	**L**	**L**	**H**	**H**	**L**	**L**	**L**
Jiang et al.^45^	**H**	**L**	**H**	**L**	**L**	**L**	**L**
Orsi et al.^46^	**L**	**L**	**L**	**L**	**L**	**L**	**L**
Peng et al.^47^	**H**	**L**	**H**	**H**	**L**	**L**	**L**
Chen et al.^55^	**L**	**L**	**L**	**L**	**L**	**L**	**L**
Jingqi et al.^48^	**L**	**L**	**L**	**H**	**L**	**L**	**L**

L = low risk; H = high risk; NA = not applicable; CEUS = contrast-enhanced ultrasound; HIFU = high-intensity focused ultrasound.

### Contrast Ultrasound Enhancement Characteristics and Quantification

Contrast-enhancement characteristics of normal uterine tissue and uterine disorders are described below and summarized in Table 2. Diagnostic accuracy, sensitivity, and specificity, NPV and PPV are shown in Table 3, if available for the cited studies.

**Table 2.** Contrast-enhanced ultrasound enhancement characteristics.

Normal uterus	Enhancement order: uterine artery and outer myometrial layer, inner myometrial layer, and endometrial layer; clear boundary endometrium and myometrium
Fibroids ≥2 cm	Initial perfusion pseudocapsule; homo- or heterogeneous enhancement entire lesion; well demarcated border between pseudocapsule and myometrium
Fibroids <2 cm	No early enhancement pseudocapsule; iso-enhancement; late phase: wash-out lesion faster than myometrium
Adenomyosis	Heterogeneous enhancement of affected myometrium
Leiomyosarcoma	Earlier enhancement feeding vessels of lesion than those of the myometrium; heterogeneous hyper-enhancement; no enhancement in center
Endometrial carcinoma	Enhancement of lesions with greater intensity than normal myometrium, with irregular, tortuous blood vessels. Wash-in and wash-out faster than normal myometrium

**Table 3.** Diagnostic accuracy of contrast-enhanced ultrasound (CEUS) with histology as reference test.

Author	Study design	Diagnosis	Number of patients	Histology	Sensitivity	Specificity	Diagnostic accuracy (%)	NPV	PPV	Remark
Zhang et al.^5^	Prospective cohort study	Fibroids	165 Fibroids	Yes	NA	NA	97	NA	NA	Nine fibroids with sarcomatous change
Song et al.^18^	Prospective cohort study	Endometrial carcinoma	35 EC	Yes	0.66	0.83	79	0.83	0.64	Averaged for disease stages 1a,b,c
Lieng et al.^19^	Prospective cohort	Endometrial carcinoma	17 EC	Yes	0.80	0.69	NR	NR	NR	
		Polyps	17 Polyps							
Liu et al.^20^	Prospective cohort study	Endometrial carcinoma	35 EC	Yes	0.86	0.85	83	NA	NA	
Liu et al.^17^	Case-control	Endometrial carcinoma	79 EC	Yes	0.82	0.73	85	0.96	0.60	
			40 Control							
Zhou et al.^21^	Prospective cohort study	Endometrial carcinoma	68 Suspect EC (26 EC)	Yes	0.77	0.74	75	0.83	0.65	2D CEUS
					0.85	0.83	84	0.90	0.76	2D + 3D CEUS
Liu et al.^22^	Prospective cohort study	Endometrial carcinoma	49 EC	Yes	0.92	0.88	83	NA	NA	Sensitivity and specificity for “*peak intensity*”
			42 Benign lesions							
Su et al.^23^	Prospective cohort study	Endometrial carcinoma	39 EC	Yes	NA	NA	83	NA	NA	Diagnostic accuracy for myometrial invasion
			42 Hyperplasia							
Zhang et al.^24^	Retrospective cohort study	Endometrial carcinoma	223 EC	Yes	0.67	0.77	NA	NA	NA	Predictive value of “*enhancement rate*” for overall survival
Green et al.^25^	Prospective cohort study	Endometrial carcinoma	93 EC	Yes	0.74	0.87	82	0.87	0.75	Values for myometrial invasion
			279 Control							
Li et al.^26^	Prospective cohort study	Fibroid/leiomyosarcoma	143 Fibroids	Yes	NA	NA	NA	NA	NA	
			4 Sarcomas							

#### Normal endometrium and myometrium

Contrast-enhancement characteristics of a normal uterus (*n* = 40) was described in one study (Liu et al.^17^).

During wash in, the uterine artery and outer myometrial layer were first enhanced followed by the inner myometrial layer and subsequently the endometrial layer. The significantly lower peak intensity of the endometrium provided a clear boundary between endometrium and myometrium. During wash-out, contrast agents subsided faster from myometrium than from endometrium.^17^

**Figure 3.**
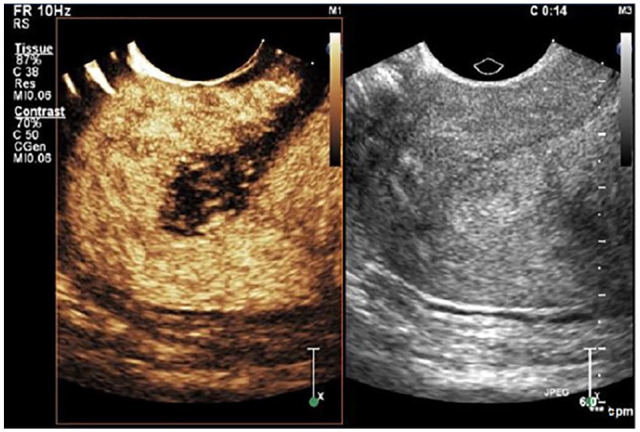
Normal uterus. *Source*. Adapted from Stoelinga et al.^49^ CEUS scan uterus: conventional gray-scale ultrasound image on the right and CEUS image on the left. CEUS image obtained 14 seconds after contrast injection shows initial enhancement of the (normal) myometrium.

Figure 3 illustrates CEUS of a normal uterus.^49^

#### Uterine disorders

*Uterine fibroids* are the most common benign tumors during the reproductive age. Although the vast majority of fibroids are well recognized by conventional ultrasound, establishing contrast-enhancement characteristics of fibroids may help discriminating other uterine disorders, such as adenomyosis and leiomyosarcomas, from fibroids. Distinctive for fibroids is the highly vascularized peripheral rim, called “pseudocapsule,” from which vessels penetrate into the center of the fibroid. Three studies described CEUS enhancement characteristics of fibroids (*n* = 348 fibroids.^5,26,34^). Fibroids enhanced earlier than the surrounding myometrium and the peak intensity differs between the two depending on the degree of fibroid degeneration. A difference was noticed between small and large fibroids, in the study of Zhang e al.^5^ with regard to enhancement of the pseudocapsule and the rest of the fibroid. Larger fibroids (>2 cm) first exhibited peripheral enhancement, followed by homogenous or heterogeneous enhancement of the entire lesion. The pseudocapsules of larger fibroids were enhanced slightly stronger than the surrounding myometrium, which resulted in a clearly demarcated border. The pseudocapsule of small fibroids (<2 cm) could not be detected in the early phase. Small fibroids exhibited synchronized iso-enhancement compared with surrounding myometrium. Most fibroids (94,5%) displayed faster wash-out of contrast than the myometrium.^30^ One study (Zhang et al.^5^) examined the diagnostic accuracy of CEUS in diagnosing fibroids compared with conventional ultrasound. Histopathology was performed as reference test on specimen obtained from operation or sonographically-guided percutaneous biopsy. This study, although of low methodological quality with a high risk of bias in patient selection, demonstrated in 96 women a diagnostic accuracy for CEUS of 97.5% for intramural fibroids and 96.3% for other types, such as submucous, subserous and cervical fibroids. Diagnostic accuracy of conventional ultrasound was significantly lower: 85.5% for intramural fibroids (*p* < .05) and 79.3% for other types of fibroids (*p* < .01).

**Figure 4.**
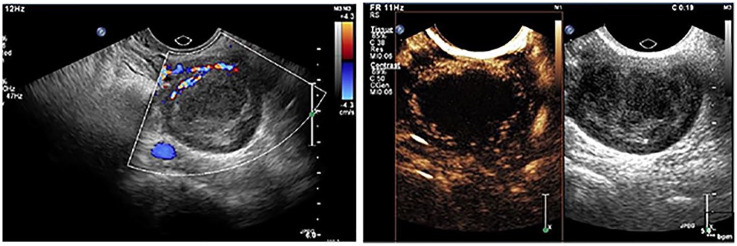
Subserosal fibroid (image made at our own institution). On the left CEUS image obtained 19 seconds after contrast injection, shows peripheral enhancement without enhancement in the central part of the fibroid. On the right conventional gray-scale ultrasound image of the same fibroid.

Figure 4 illustrates CEUS of a subserosal fibroid.^49^

Aside pathology, currently accurate diagnostic tools for *leiomyosarcomas* are lacking as there are no specific symptoms discriminating malignant leiomyosarcomas from benign (atypical) fibroids. Although a leiomyosarcoma is rare, it is highly aggressive and contributes to a significant proportion of uterine cancer deaths.^4,50^ Given the aggressiveness of the disease, the risk of tumor cell dissemination in case of inadvertent morcellation^50,51^ and the lack of reliable diagnostic criteria, there is a need for an imaging technique able to detect this malignancy. Two studies evaluated women with different types of fibroids, including leiomyosarcomas.^5,26^ Zhang et al.^5^ evaluated 96 women with fibroids (also discussed under “uterine fibroids”) and leiomyosarcomas using CEUS and conventional ultrasound, with histology (after surgery or biopsy) as reference test. Li et al.^26^ performed CEUS in 147 patients pre-operatively, with post-operative histology as reference test. The contrast-enhancement pattern was similar for all leiomyosarcomas (*n* = 9 and 4, in studies by Zhang et al.^5^ and Li et al.^26^ respectively). Sarcomatous lesions showed a heterogeneous hyper-enhancement due to increased blood flow, whereas fibroids (*n* = 156 and 143 respectively) showed homogeneous enhancement.^5^ Seventeen fibroids showed signs of degeneration, of which eight benign and nine malignant. The benign fibroids showed no contrast perfusion in the degenerated area while other parts of the tumors showed the same perfusion as non-degenerated fibroids. The margin of sarcomatous lesions was poorly defined, while fibroids could be well demarcated. Feeding vessels of sarcomatous lesions enhanced earlier than those of the myometrium. By this feature, CEUS was able to detect all nine sarcomas, while conventional US was able to detect only two.^5^ Neither studies reported on perfusion parameters or diagnostic accuracy, possibly due to the low number of leiomyosarcomas, yet a diagnostic accuracy of 97% for leiomyosarcoma can be deduced for the study of Zhang et al.,^5^ but not for the study by Li et al.^26^

*Adenomyosis* is characterized by benign growth of endometrial tissue into the myometrium. The diagnosis is often missed due to its diffuse character^30,52^ which often requires an additional MRI scan.^53^ A recent systematic review described changes in microvasculature of adenomyotic tissue in comparison with normal myometrium due to increased angiogenesis.^54^ Therefore, CEUS could theoretically be used to diagnose adenomyosis, however, no studies are yet published on this topic. To date four studies performed CEUS in women who underwent a minimally invasive treatment (HIFU and microwave ablation) of adenomyosis.^30,37,40,48^ The contrast-enhancement characteristics before ablation were shortly described by Xu et al.^40^ as “synchronous enhancement of the entire lesion” (45 cases) and “slow filling from the periphery to the center of the lesion” (21 lesions).

*Endometrial carcinoma* is the most common gynecological malignancy.^18^ This is reflected in the higher number of studies evaluating CEUS for this malignancy: one case-control^17^ and seven prospective cohort studies,^18,20-25^ with histology-proven endometrial carcinoma. These studies did show different enhancement patterns between malignant and healthy tissue. The principal findings were that enhancement of malignant lesions was in general earlier and with greater intensity than that of normal myometrium and endometrium.^17,18,20,21,25^ Compared with surrounding tissue, contrast-enhancement was first observed in 57% to 82% of the tumors,^18,20,25^ with most lesions (61%) being hyper-echogenic, 27% was iso-echogenic, and 11% hypo-echogenic.^17^ In 77% the feeding vessels of the tumor were enhanced first and then branched into the endometrial cancer. In the other 23% signals were first visualized in the central portion of the tumor.^18^ Inhomogeneous enhancement was observed in 66% of the lesions while 34% showed homogeneous enhancement (Table 2). Importantly, it was shown that endometrial carcinoma had significantly lower perfusion time parameters and higher intensity parameters compared with benign endometrial lesions.^22^ The diagnostic value of 3D-CEUS was also demonstrated. In benign lesions, 3D-CEUS showed straight blood vessels of regular shapes near the lesion and sparse blood vessel distribution within the lesion. In malignant lesions, 3D-CEUS revealed tortuous, irregular blood vessels, twisted into groups.^21^

Contrast enhanced color- and power Doppler was applied in case of *polyps* (*n* = 17) and histology proven endometrial cancer The authors demonstrated that after the injection of intravenous contrast the pulsatility index and resistance index were significantly lower in malignant endometrial lesions.^19^ This could be of value for discriminating between benign and malignant endometrial pathology.

Determining the degree of myometrial invasion is important for the staging procedure, as this may affect surgical strategies. The average accuracy of CEUS as diagnostic tool for staging endometrial carcinoma is 81%, with an average sensitivity of 0.78 and specificity of 0.81.^17,18,20-25^ The numbers of the individual studies are shown in Table 3. The additional value of combined 2D- and 3D-CEUS imaging (diagnostic accuracy 84%) over 2D-CEUS alone (diagnostic accuracy 75%) was demonstrated in 68 patients with clinically suspected endometrial carcinoma, of which 26 cases were confirmed by histology.^21^ Finally, the perfusion parameter “enhancement rate” (dB/s) determined pre-operatively was shown to be an independent predictor for both recurrence of endometrial carcinoma (hazard ratio 1.7; 95% CI 1.0–7.7; *p* < .05) and overall survival (hazard ratio 2.0; 95% CI 1.0–7.8; *p* < .05) in a retrospective study with 223 patients.^24^

### Monitoring and Enhancing Effectiveness of Minimally Invasive Therapies

#### CEUS for monitoring ablation therapies

The predictive value of quantitative perfusion parameters obtained by CEUS for the therapeutic response to HIFU was assessed by Wang et al.^39^ They showed that fibroids with lower perfusion time parameters and higher intensity parameters, that is, “quick-rise-quick-decline” blood flow profile, had poor ablation efficacy. Table 4 provides an overview of the studies using CEUS pre- and post-treatment to image changes in perfusion.

**Table 4.** Contrast-enhanced ultrasound (CEUS) for monitoring and enhancing minimally invasive therapy.

Monitoring post-therapy						
Reference	Treatment	Study design	*N*	CEUS (pre, per, post)	MRI (pre, per, post)	Result study/outcome CEUS
Dorenberg et al.^27^	UAE fibroids	Safety/feasibility	10	Pre	Pre	Feasibility of CEUS during UAE. Outcome measured with CEUS and compared with MRI: 1 day post-UAE 9/10 pts: MRI = CEUS; 3 months post-UAE 10/10 pts: MRI = CEUS.
				Per (directly after UAE)	Post (1 day, 3 months)	
Zhou et al.^28^	HIFU fibroids	Prospective cohort study	64	Post (1 week, 1, 3, 6, and 12 months)	Post (1 week)	Utility of CEUS for early post-HIFU efficacy. Outcome measured with CEUS and MRI; “Got same results with CEUS as with MRI.”
Sconfienza et al.^29^	SUFE fibroids	Safety/feasibility	12	Pre	Post (6 months)	Feasibility of CEUS during SUFE. Outcome measured with CEUS and MRI; “CEUS findings similar to dynamic MRI”
				Per		
				Post (1 and 6 months)		
Wang et al.^30^	HIFU adenomyosis	Safety/feasibility	12	Pre	Pre	Feasibility HIFU of adenomyosis. Outcome measured with CEUS.
				Post (1 hour)		
Wang et al.^31^	Microwave fibroid	Prospective cohort study	29	Pre	No	Accuracy of CEUS in assessing MWA efficacy. Outcome measured with CEUS and conventional US; Pearson *r* = 0.997.
				Per (directly after MWA)		
				Post (12–24 hours)		
Wang et al.^32^	HIFU fibroids	Safety/feasibility	76	Pre	Pre	Feasibility USgHIFU of submucosal fibroids. Outcome measured with CEUS and MRI; CEUS vs. CEUS; MRI vs. MRI.
				Per (directly after HIFU)	Post (1, 3, 5, and 12 months)	
				Post (1, 3, 6, and 12 months)		
Lei et al.^33^	Microwave fibroid	Prospective cohort study	36; *18 fibroid*	Post (7 days)	Post (7–10 days)	Accuracy of CEUS in assessing MWA efficacy. Outcome measured with CEUS and MRI; ICC 0.99.
Wang et al.^35^	HIFU fibroids	Prospective cohort study	67	Per (directly after HIFU)	Post (directly after HIFU; 1, 3, 6, and 12 months)	Accuracy of CEUS in assessing HIFU efficacy. Outcome measured with CEUS and MRI; ICC 0.91.
				Post (1, 3, 6, and 2 months)		
Xia et al.^36^	Microwave fibroid	Prospective cohort study	88	Per (directly after MWA)	Pre	Optimized MWA parameters based on MRI before treatment. Outcome measured with CEUS.
Wang et al.^39^	HIFU fibroids	Retrospective cohort study	263	Pre (directly before HIFU)	Pre, Post (1 day)	Predictive value of CEUS perfusion parameters on HIFU outcome. Correlation CEUS perfusion parameters pre-HIFU with non-perfused volume ratio post-HIFU.
				Per		
Henri et al.^34^	UAE fibroids	Prospective sohort study	40	Pre	Pre, Post (6 and 12 months)	CEUS is feasible and useful to understand fibroid vascularization and monitoring embolization; its correlation with MRI is good.
				Post (directly after AUE, 6 and 12 months)		
Yu et al.^37^	Microwave adenomyosis	Prospective cohort study	278	Per (directly after MWA)	Pre	Feasibility MWA of adenomyosis. Outcome measured with CEUS and MRI.
					Post (3 days)	
Zhang et al.^38^	Microwave fibroid	Prospective cohort study	120; *60 fibroid*	Pre (1 week)	Pre (1 week), Post (6, 12, and 24 months)	Accuracy of CEUS in assessing MWA efficacy. Outcome measured with CEUS and MRI; “CEUS is advantageous over MRI.”
				Post (6, 12, and 24 months)		
Xu et al.^40^	Microwave adenomyosis	Prospective cohort study	66	Pre	Pre	Accuracy of CEUS in assessing MWA efficacy. Outcome measured with CEUS and MRI; *R* = 0.81 for ablation rates
				Post (1–2 days)	Post (1–2 days)	
Enhancing minimal invasive therapy						
Dorenberg et al.^42^	CEUS-enhanced UAE fibroid	Retrospective cohort study	30	Per (directly after UAE)	Pre	Enhanced UAE by intraprocedural CEUS. Outcome measured with CEUS and MRI.
					Post (3 and 12 months)	
Peng et al.^41^	Intra-procedure CEUS HIFU fibroids	Retrospective cohort study	291	Pre	Pre	Utility of intra-procedural CEUS during HIFU. Outcome measured with CEUS and MRI; “results from CEUS correlated well with results from MRI.”
				Per (10 minutes prior to HIFU)	Post (1 day)	
				Post		
Jiang et al.^45^	CEUS-enhanced HIFU fibroid	Randomized controlled trial	80	Per (MB infusion 5 minutes prior to HIFU)	Pre	Feasibility of CEUS-enhanced HIFU. Outcome measured by MRI.
				Post (directly after HIFU)	Post (1 day)	
Cheng et al.^43^	Intra-procedure CEUS HIFU fibroids and adenomyosis	Retrospective/safety cohort study	2604	Per (directly after HIFU)	Pre	Safety of intra-procedural CEUS during HIFU. Outcome measured with CEUS during HIFU and MRI post-therapy.
					Post (1 day)	
Peng et al.^47^	Intra-procedure CEUS HIFU fibroids	Prospective cohort study	68	Per (8 minutes prior to HIFU)	Pre	Utility of intra-procedural CEUS during HIFU. Outcome measured with CEUS and MRI; *R* = 0.96 for NPV.
				Post (directly after HIFU)	Post (1 day)	
Orsi et al.^46^	CEUS-enhanced HIFU fibroid	Randomized controlled trial	33	Pre	Pre (1 week)	Feasibility of CEUS-enhanced HIFU. Outcome measured by MRI.
				Per (MB infusion 15 seconds prior to HIFU)	Post (1, 3, 6, and 12 months)	
				Post (directly after HIFU)		
Isern et al.^44^	CEUS-enhanced HIFU fibroid	Retrospective cohort study	319	Per (MB infusion 2 minutes prior to HIFU)	Pre	Safety of CEUS-enhanced HIFU. Outcome measured by MRI.
					Post (directly after HIFU)	
Jingqi et al.^48^	CEUS-enhanced HIFU adenomyosis	Safety study cohort study	102	Per (1 or 10 minutes prior to HIFU)	Pre	Safety of CEUS-enhanced HIFU. Outcome measured by MRI.
					Post (1 day)	
Chen et al.^55^	CEUS-enhanced HIFU fibroid	Prospective cohort study	120	Per (MB infusion 6/10 minutes prior to HIFU)	Pre	Feasibility of CEUS-enhanced HIFU. Outcome measured by MRI.
				Post (directly after HIFU)	Post (1 day)	

*HIFU* is a minimally invasive therapy to ablate fibroids and adenomyosis. Three studies described the ability of CEUS to detect non-perfused ablation areas in treated fibroids^28,32,35,39^ and two studies in adenomyosis.^30,48^ These studies also performed MRI as reference test, however only one study compared CEUS and MRI data and found an ICC of 0.910 (*p* < .01).^35^ Two retrospective^41,43^ and one prospective study^47^ examined the safety of CEUS as intra-procedural, real-time technique for US-guided HIFU to assess ablation results. Residual CEUS-enhancement was observed in 15% to 35% of the treated lesions and an additional HIFU treatment was then performed in the same session. Apart from monitoring, CEUS can also be used to enhance effectiveness of HIFU when applied *just before* treatment. It is thought to work via cavitating microbubbles during HIFU exposure, which may mechanically destruct the fibroid and further increase the temperature for ablation therapy. Five studies, one retrospective^44^ and four prospective studies^45,46,48,55^ investigated this enhancing potential of CEUS on HIFU treatment. However, all studies choose different time points of injecting SonoVue, varying from 1 to 10 minutes prior to HIFU treatment. Elimination half-life of SonoVue is approximately 6 minutes,^56^ these studies were therefore performed under different blood concentrations of SonoVue. Most important findings of these studies were shorter insonication time required to ablate 1 cm^3^ of fibroid to reach massive grayscale changes, and less energy applied in the presence of microbubbles. Results on fibroid shrinkage on the long-term did not differ.

*Microwave ablation* is similar to HIFU, that is, an ablation technique that aims at heating fibroid tissue. Where HIFU works within diagnostic frequencies (0.6–5.0 MHz), microwave ablation works with electromagnetic waves (900–2450 MHz) and is typically used to ablate larger tumors (>3.0 cm).^57^ Four studies demonstrated the use of CEUS for monitoring microwave ablation of fibroids,^31,33,36,38^ and two studies for monitoring this treatment of adenomyosis.^37,40^ Quantitative analysis demonstrated good agreement between contrast-enhanced MRI and CEUS for detecting non-perfused volume after microwave ablation of fibroids (ICC = 0.991)^33^ and ablation rate in adenomyosis (*R* = 0.81).^40^

#### CEUS for monitoring uterine artery embolization

*Uterine artery embolization* (UAE) is a minimally invasive technique injecting embolization particles under angiographic guidance to occlude the uterine artery and thereby blood supply to the fibroid. Three pilot studies demonstrated feasibility of CEUS to image the degree of fibroid perfusion directly after UAE in the angiography room. All three studies showed good agreement between CEUS and MRI results.^27,29,34^ Successful application of CEUS *during* UAE was also described in a retrospective study (*n* = 30). In five cases the endpoint of embolization was adjusted based on findings with CEUS, that is, embolization was continued.^42^

### Contrast Agents and Adverse Events

In general, adverse reactions to ultrasound contrast agents in humans are rare, usually transient and of mild intensity. The incidence of severe hypersensitivity or anaphylactic reactions is less than 0.002%, which is lower than current X-ray agents and comparable with MR contrast agents.^58^ SonoVue was used as contrast agent in all of the included studies. The administered dose ranged from 1.0 to 4.8 mL, the most commonly used dosage was 2.4 mL. No serious adverse events were reported in any of the discussed studies. In the studies where CEUS was used to monitor or enhance effectiveness of minimally invasive therapies common ablation-related side-effects were mentioned, such as discomfort hot skin sensation and pain in treated region. The side-effects were transient and disappeared 1 to 4 hours post-procedure, all pain scores were mild (<4).^20,27-39,41,43-48,55^ The differences in side-effects between “HIFU” and “HIFU + CEUS” are listed in Supplemental Appendix B.

## Discussion

Contrast-enhanced ultrasound in gynecology has gained more and more interest over the last two decades. We provided an extensive systematic review to describe CEUS enhancement characteristics for specific myometrial and endometrial disorders that may serve as a basis for potential diagnostic differentiation of uterine fibroids, adenomyosis, leiomyosarcoma, and endometrial carcinoma. Yet conclusive evidence still has to be provided by well-designed clinical trials. This review does demonstrate that CEUS can readily be used to monitor effectiveness of minimally invasive therapies. CEUS may also be used to stimulate the effectiveness of these minimally invasive therapies, however, the added value on the longer term is not yet demonstrated. The safety of this intraprocedural CEUS in terms of side-effects seems not be affected, though this needs to be carefully evaluated in future studies. This was recently also concluded in a review specifically about CEUS for monitoring minimally invasive treatments for uterine fibroids.^59^

There are established methods for diagnosing several uterine disorders, and one may question the added value of CEUS for example for fibroids, which are in general well recognized on grayscale ultrasound. Adenomyosis, however, remains a challenging disorder to diagnose based on grayscale ultrasound. Another challenge is the diagnosis of leiomyosarcomas as the clinical presentation of uterine sarcomas is nonspecific and the ultrasound characteristics of grayscale ultrasound resemble those of fibroids. In order to achieve accurate differentiation between different disorders using CEUS it is important to document the contrast-enhancement characteristics of fibroids as well as other pathologies in a structured and standardized manner. Up till now, enhancement characteristics have been described in the researchers sole discretion. This is also reflected in Table 2, and unfortunately this does not provide the necessary diagnostic information when assessing a patients CEUS scan for adenomyosis or a malignancy.

In general, diagnosis of uterine disorders and monitoring effectiveness of minimally invasive therapies can typically be performed using dynamic MRI, as this reference test provides good quality information on morphology, size, and vascularization. In clinical practice, CEUS may be added as second diagnostic imaging technique after the initial grayscale ultrasound and prior to an MRI scan—which typically has long waiting lists—or in medical centers where MRI is not available.^29^ In order for CEUS to become a clinically established imaging technique and potentially an alternative for MRI, diagnostic accuracy should be determined not only by histology, but also by quantitative comparison with MRI. Remarkably, none of the studies that used CEUS as a diagnostic tool performed an MRI, all used histology as reference test. Fifteen studies performed MRI as post-treatment imaging modality, of which six studies described that results obtained with both CEUS and MRI were similar^27-29,38,41,45^ and four studies made a statistical comparison of CEUS with MRI^33,35,40,47^ in gynecology. In the characterization of non-gynecology malignancies, for example focal hepatic lesions and recognizing malignancies, there are studies showing a similar-to-higher accuracy of CEUS compared with MRI.^60,61^ Notably, CEUS has several advantages over MRI: (1) imaging in real-time, allowing continuous assessment of the enhancement period, whereas MRI scans can only be made at distinct time points; (2) ultrasound contrast agents remain intravascular, unlike MRI contrast agents, providing information specifically on vascular flow; (3) high spatial resolution, ability to imaging the microvasculature; (4) the option to apply CEUS in out-patient clinic; (5) lower cost than MRI and CT.^62^

### Limitations

Despite the high potential of CEUS, the methodological quality of the included studies could not provide conclusive support for current implementation of CEUS in daily gynecological practice. As mentioned before, the role of CEUS *has been established in other fields of specialty.* This might be taken as “fait accompli” by some groups, who have put CEUS into use in gynecology without the need for setting up a clinical study aimed at establishing the role of CEUS with a consistent report of enhancement characteristics and/or statistical comparison with MRI.

The included studies may represent a selection bias as most studies did not report on inclusion and exclusion criteria and the number of excluded patients. In addition, most studies had small sample sizes and patients were selected based on previous diagnosis of uterine pathology. This could mean that the diagnostic value of CEUS may be overestimated. Additionally, by far most of the referenced studies were conducted in China, a country with a wide use of CEUS in various clinical scenarios.

A note of interest is that we cannot exclude a possible overlap between included patients in some of the papers. Liu et al.^17^ included patients with histology-proven endometrial carcinoma between January 2008 and April 2011. Whereas the study by Liu et al.^22^ published in 2016 included patients between January 2010 and March 2014. The retrospective study by Peng et al.^47^ on intraprocedural CEUS during HIFU may have described the results from a number of the same patients that were included in a prospective study by the same group in 2014.^45^

## Conclusion

This review demonstrates the limited number of methodological high-quality clinical studies and structural reports on CEUS application in gynecology. CEUS obtained different contrast-enhancement patterns between malignant tissue (leiomyosarcomas and endometrial carcinoma) and healthy or benign tissue (normal myometrium, fibroids or adenomyosis); providing a first base for potential diagnostic differentiation in gynecology. In addition, the results show that it is also possible to determine the degree of myometrial invasion in case of endometrial carcinoma using CEUS. The effectiveness of minimally invasive therapies for uterine disorders can safely and accurately be assessed with CEUS.

In conclusion, CEUS is a promising technique and it is worth further exploring its full potential for gynecology by designing innovative and methodologically high-quality clinical studies.
